# A rare early-onset neonatal case of Birk-Barel syndrome presenting severe obstructive sleep apnea: a case report

**DOI:** 10.3389/fmed.2023.1180337

**Published:** 2023-06-08

**Authors:** Qian Zhang, Zhen Qin, Ruolan Hu, Yifei Li, Fan Yang, Jinrong Li

**Affiliations:** ^1^Key Laboratory of Birth Defects and Related Diseases of Women and Children of MOE, Department of Pediatrics, West China Second University Hospital, Sichuan University, Chengdu, Sichuan, China; ^2^Department of Anesthesiology, West China Hospital, Sichuan University, Chengdu, China

**Keywords:** *KCNK9*, OSA, Birk-Barel syndrome, TASK3 channel, case report

## Abstract

**Background:**

Birk-Barel syndrome, also known as KCNK9 imprinting syndrome, is a rare fertility disorder. And the main clinical manifestations include congenital hypotonic, craniofacial malformation, developmental delay, and intellectual disability. Generally, such patients could be diagnosed beyond the infant period. Moreover, the delayed diagnosis might lead to a poor prognosis of rehabilitation therapy. However, neonatal obstructive sleep apnea (OSA) was seldom reported in Birk-Barel syndrome. Here, we reported a severe neonatal OSA case induced by Birk-Barel syndrome, resulting in an early diagnosis with improved outcomes by integrative management.

**Case presentation:**

The proband was a neonate presenting with recurrent severe OSA, with craniofacial deformity and congenital muscle hypotonia. Bronchoscopy examinations indicated a negative finding of pharyngeal and bronchus stenosis, while laryngomalacia had been observed. Whole exon sequencing demonstrated a c. 710C>A heterozygous variant resulting in a change of amino acid (p.A237D). This variant resulted in a change of amino acid sequence, affected protein features and changed splice site leading to a structural deformation in KCNK9 protein. This p.A237D variant also affected the crystal structure on the p.G129 site. Additionally, we used the mSCM tool to measure the free energy changes between wild-type and mutant protein, which indicated highly destabilizing (−2.622 kcal/mol).

**Conclusion:**

This case report expands the understanding of Birk-Barel syndrome and indicates that OSA could serve as the on-set manifestation of Birk-Barel syndrome. This case emphasized genetic variants which were associated with severe neonatal OSA. Adequate WES assessment promotes early intervention and improves the prognosis of neurological disorders in young children.

## 1. Introduction

Obstructive Sleep Apnea (OSA) is a sleep-related condition characterized by upper airway obstruction and is commonly observed in adults, particularly in obese individuals. However, the prevalence of OSA in children is estimated to be around 1–5% (1). OSA is rarely diagnosed in neonates due to a lack of clear diagnostic criteria and normative records, leading to its underdiagnosis (2). Central apnea attacks are often considered the most common disorder during neonatal disease management, making OSA easier to overlook. OSA can be challenging to distinguish from other disorders, even with the use of invasive and noninvasive examinations (1). Neonatal OSA is most commonly observed in individuals with craniofacial malformations and can also be attributed to neuro-muscular disorders, bronchopulmonary dysplasia, prematurity, adverse prenatal exposure, and overweight newborns (1). With the advancements in Next Generation Sequencing (NGS) technology, whole exome sequencing (WES) has increased our understanding of the genetic basis of OSA (3). In addition to chromosomal abnormalities, several inherited genetic disorders, such as spinal muscular atrophy (*SMN1* gene variant), CHARGE syndrome (*CHD7* gene variant), *ADRB2* and *EDN1* variants, have been linked to neonatal OSA. Timely diagnosis and appropriate treatment of OSA can help prevent unexpected neonatal sudden death (4).

Birk-Barel syndrome is a rare genetic disorder that presents with severe hypotonia, developmental abnormalities, and other symptoms in toddler and school-age children. The delayed diagnosis of this condition is often a result of its complex and varied symptoms. While OSA has been reported in older children with Birk-Barel syndrome, it has not been previously documented in neonates. Birk-Barel syndrome, also known as KCNK9 imprinting syndrome, is caused by mutations in the *KCNK9* gene located on chromosome 8q24.3. This gene is part of the double-pore domain potassium channel gene family and encodes the K2P9.1 (TASK3) protein, which has a high expression in neurons and plays a role in the migration of neurons in the cerebral cortex and vertebral bodies (5). The majority (80%) of Birk-Barel syndrome cases are caused by a maternal genetic variant, while the remaining 20% are de novo variants (6). However, the maternal allele carrier usually presents with a normal phenotype. The clinical manifestations of Birk-Barel syndrome include central hypotonia, palatal abnormalities, sacral recess, facial deformities, developmental delay, feeding difficulties, and other symptoms. In this case report, we present a rare and early-onset neonatal case of Birk-Barel syndrome with the *KCNK9* p.A237D variant, presenting with severe and persistent OSA. This is the youngest patient reported with Birk-Barel syndrome to date. Rapid WES analysis identified the *KCNK9* variant, leading to improved diagnosis and treatment. This case highlights the importance of WES in the management of neonatal OSA and the benefits of early intervention in improving the prognosis of neurological disorders in young children.

## 2. Clinical description and molecular results

### 2.1. History of illness and physical examination

This research has been approved by the Ethics Committee of the West China Second Hospital of Sichuan University (Approval No.2014-034). Informed consent was obtained from the patient's parents before performing WES and for the inclusion of the patient's clinical and imaging details in subsequent publications.

The proband was a male neonate who was born full-term at 38+3 gestational weeks via cesarean delivery, with a birth weight of 3,500 g. The neonate scored 10/10 on the Apgar scale at 1, 5, and 10 min after birth. However, 17 min after birth, he presented with severe cyanosis and difficulty breathing, causing a significant drop in his blood oxygen saturation. Acute respiratory distress syndrome (ARDS) and pulmonary dysfunction were suspected and treated with invasive measures, including the administration of three doses of pulmonary surfactant, antibiotics, and other supportive therapies. Although initial treatment partially relieved the neonate's symptoms of hypoxia, recurrent apnea was observed, particularly during sleep, which resulted in a further reduction in blood oxygen saturation. Despite administering intravenous caffeine to stimulate the central nervous system, the apnea symptoms were not improved.

**Figure 1:**
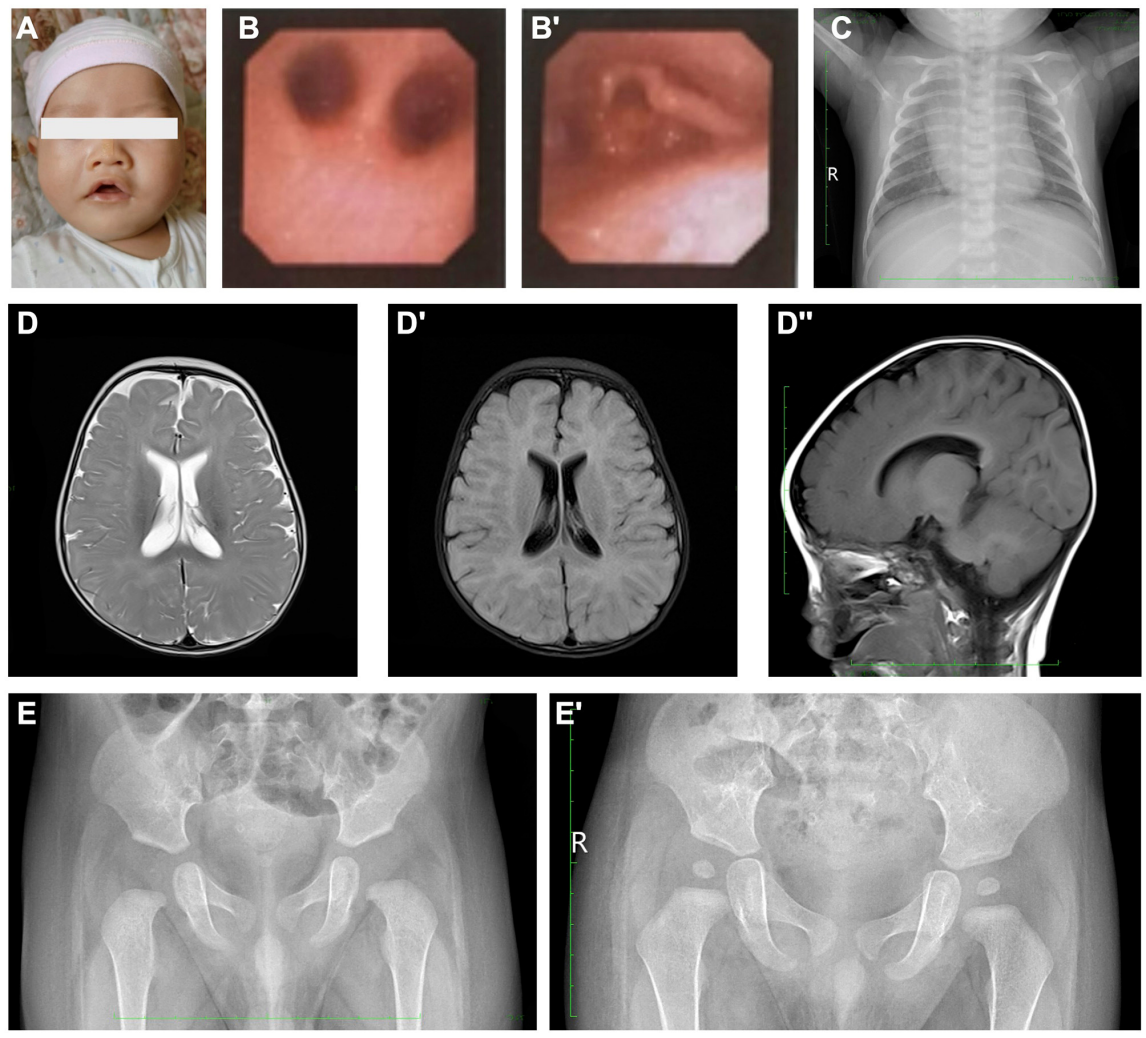
Clinical images presentation of the proband. **(A)** Abnormal facial appearance of the proband. **(B, B')** Bronchoscopy examinations indicated a negative finding of pharyngeal and bronchus stenosis, while only laryngomalacia could be observed. **(C)** Chest X-ray of the proband. **(D–D”)** Cerebral MRI demonstrated normal morphology and signaling. **(E, E')** Pelvic X-ray examination. Image **(E)** was taken at 3-month-old, and **(E')** was taken at 1.5-year-old.

The proband was subjected to a physical examination that revealed an increased respiratory rate of 60 breaths per minute. The blood oxygen saturation was 65% (without oxygen supplementation) prior to intubation. The neonate demonstrated a poor response to external stimuli and exhibited a type of craniofacial deformity, characterized by bilateral temporal narrowing, maxillary protrusion, mandibular retraction, a broad nasal tip, a short and wide philtrum, a drooping upper lip, a lower lip that was shorter than the upper lip, and an altered mouth opening shape (Figure 1C). The neonate's lips were cyanotic and breath sounds were rough in both lungs, with wet rales audible. The heart's boundary was within normal range, and heart sounds were strong with a regular rhythm. No murmurs were recorded. The abdomen was soft, the liver was 1 cm below the subcostal margin and 1–2 cm below the xiphoid process, the texture was medium, and the spleen was not palpable subcostally. An evaluation of extremity muscle tone revealed hypotonia.

The parents of the proband reported no history of pulmonary developmental disorders, congenital disabilities in the respiratory system, neurological impairment, or developmental disabilities among their family members. The mother had a history of five pregnancies, with two resulting in miscarriages and a diagnosis of a hydatidiform mole during the second pregnancy. The fourth pregnancy involved the use of an embryo transfer technique, but the embryo ceased development at 8 weeks and was found to have a trisomy of chromosome 2 with 14% chimerism upon analysis.

### 2.2. Imaging and laboratory examinations

The initial laboratory results showed routine blood cell tests and blood gas analysis within normal limits. The results of hepatic and renal function tests showed no significant findings. However, the blood ammonia level was significantly elevated to 69 umol/L. At the same time, the cerebrospinal fluid examination, respiratory pathogen detection, and urine organic acid test were also performed and showed negative results. Due to recurrent apnea, the patient underwent bronchoscopy exams, which revealed no pharyngeal and bronchus stenosis (Figure 1B), and only laryngomalacia was observed (Figure 1B'). The chest X-ray examination after 10 days of invasive ventilation and antibiotics administration showed a typical presentation (Figure 1A). The echocardiography revealed a normal cardiovascular structure except for the presence of a patent foramen ovale. The cerebral MRI scan showed a typical neonatal presentation and signal pattern without significant lesions (Figures 1D–D”). Furthermore, the long-term video electroencephalogram, peripheral nerve function test of upper and lower extremities, and maxillofacial and neck CT failed to reveal any positive findings. The pelvic X-ray showed normal structure during the neonatal period and at the 1.5-year follow-up (Figures 1E, E').

### 2.3. Molecular results

**Figure 2:**
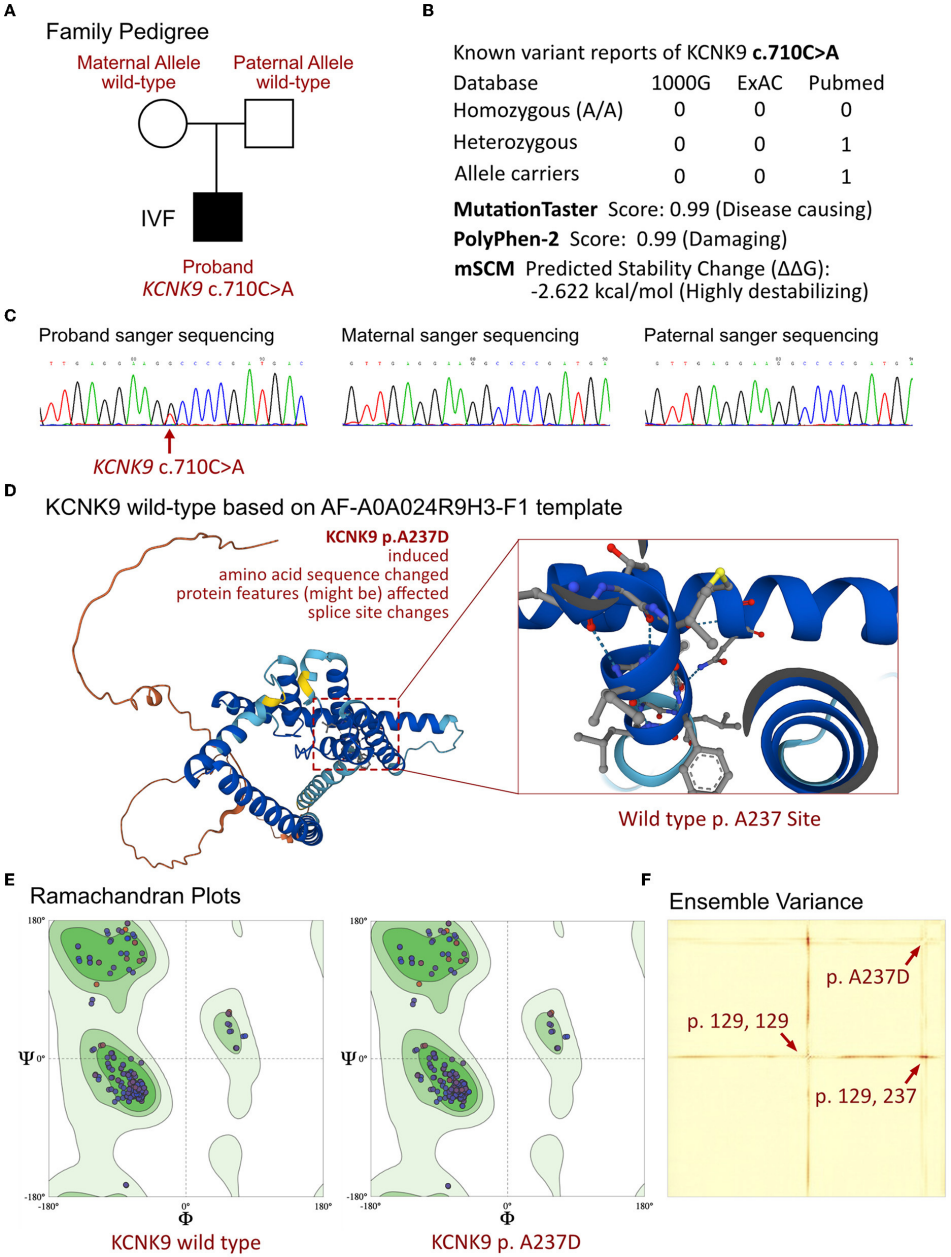
Molecular analysis of KCNK9 variants. **(A)** Family pedigree of the proband. **(B)** The prevalence of *KCNK9* variant identified in the proband. **(C)** The sanger sequencing validation of the family. **(D)** Molecular structure based on AF-A0A024R9H3-F1 templet. **(E)** Ramachandran plots of KCNK9 protein between wild-type and p.A237D variant. **(F)** Ensemble variance demonstrated the changes on protein structural stability.

The proband, who presented with neonatal severe recurrent apnea, facial deformity, and severe hypotonia, was strongly suspected of having a significant genetic disorder. WES was performed on the proband and his parents, and a heterozygous mutation in the *KCNK9* gene was identified in the proband (Figure 2A). The variant, c.710C>A, resulted in a change of the amino acid from alanine to aspartic acid (p.A237D) and was considered a de novo mutation, as it was not present in the parents. No other related variants that have been linked to severe apnea, respiratory failure, metabolic disorders, birth defects, or neurological developmental dysfunction were found in the family. The frequency of this mutation in the population is not recorded in the ExAC or 1000G databases. Further analysis revealed that the p.A237D variant affected the crystal structure on the p.G129 site and resulted in a loss of protein function (Figure 2C). The variant was considered a disease-causing mutation with a probability of 0.99 according to MutationTaster analysis and a damaging protein structure score of 0.99 from Polyphen-2 analysis. The mSCM tool was used to measure the free energy changes between the wild-type and mutant protein, indicating a highly destabilizing change of −2.622 kcal/mol (Figure 2). Previous research has shown that the p.A237D mutation of the *KCNK9* gene results in a decreased sensitivity of the TASK3 channel to extracellular pH and activated G protein-coupled receptors, which can alter the regulation of the channel and lead to various diseases.

### 2.4. Final diagnosis, treatment, and follow-up

The patient was diagnosed with Birk-Barel syndrome 3 weeks after birth due to his clinical presentations and genetic results. During his hospitalization, he received treatment including non-invasive ventilation with continuous passive airway pressure (CPAP) support, antibiotics, nutrient administration, and swallowing function training. After being discharged, he continued to receive high-flow oxygen inhalation to prevent recurrent episodes of OSA and sudden infant death.

During the follow-up, we found majority of milestones are significantly backward, including raise up head at 6 months old, sit alone at 11 months. The Gesell assessment at the age of 6 months old indicated that severe defects in adaptability, motor skills (DQ 27), fine motor skills, language, and individual society. The patient was also referred for early developmental support/special education, including physical therapy, occupational therapy, speech therapy, and cognitive therapy. With the optimal and timely intervention, the patient was able to maintain essential physical growth and improved neuromotor development (DQ 50 of Gesell assessment at 18 months old), however, muscle hypotonia remained a significant issue.

## 3. Discussion

The KCNK9 gene, located on chromosome 8, is an imprinted gene that has been linked to developmental disorders. The disease caused by dysregulation of *KCNK9* has been described as a developmental disorder. Studies have indicated that variants of *KCNK9* can impair cellular proliferation and lead to decreased TASK3 channel activity in TASK3 gene knockout mice, resulting in cognitive dysfunction. The TASK3 channel, encoded by the *KCNK9* gene, regulates resting membrane potential and influences action potential duration and neuronal firing frequency. These channels are highly expressed in neurons, particularly in cerebellar neurons (7), and play an essential role in the development of the central nervous system and functional maturation. The TASK3 channel is sensitive to extracellular pH, neurotransmitters, and volatile anesthetics (7). The reported variants of *KCNK9* have varying effects on channel function, including alterations in current potential, Gαq, and extracellular acidification dysregulation. For example, the p.G236R mutation reduces the outward current of the TASK3 channel by approximately 80%, while the p.A237D variant reduces sensitivity to extracellular pH and activated GPCRs, but fails to significantly reduce the outward current compared to the wild-type TASK3 channel. This suggests that different variants of *KCNK9* may result in different clinical manifestations.

According to a literature review, the main symptoms of Birk-Barel syndrome are congenital hypotonia, craniofacial malformations, developmental delays, and intellectual disability. Cousin et al. (6) documented 47 cases with 19 unique KCNK9 variants, of which 15 new variants exhibited phenotypic characteristics similar to previously reported cases with p.G236R variants. They proposed that there are two mutation hotspots in the KCNK9 gene, located at the p.G236 codon and the newly discovered p.R131 codon. The p.A237D mutation was first reported in 2020, and the primary symptom associated with this mutation was described as axonal peripheral polyneuropathy (8). Among all reported cases, there is only one reported case of Birk-Barel syndrome, with apnea being the initial clinical manifestation. However, the variant site for this patient was not addressed. Our proband is the youngest patient to receive a confirmed diagnosis of Birk-Barel syndrome through rapid familial NGS-based WES. Unlike other reported cases, the proband did not exhibit palatal abnormalities, sacral recess, transient neonatal hypoglycemia with hyperinsulinemia, joint contractures, myoclonus, or seizures. The dominant clinical feature of this case was severe OSA, which was attributed to the hypotonia of the muscles around the trachea and was identified as the KCNK9 c.710C>A p.A237D variant. In addition, special facial dysplasia (small jaw or cleft palate), which is very similar with Pierre Robin sequence at an early stage of life, and may cause the tongue to block the airway, also resulting in OSA. Unlike the Pierre Robin sequence, Birk-Barel syndrome also combined with other abnormal neurological developmental disorders, especially hypotonia. What is more, the special facial abnormalities of Birk-Barel syndrome will gradually appear after the neonatal period including special leg length, micrognathia and asymmetric ears, malocclusion, bitemporal retrusion, and nasal deviation. As such, Birk-Barel syndrome should be considered for patients with severe neonatal OSA, abnormal neurological developmental disorders especally hypotonia and special facial features, and WES analysis can help diagnose rare inherited neonatal diseases in a timely and accurate manner.

Currently, there is no established treatment guideline for Birk-Barel syndrome. Research has shown that three non-steroidal anti-inflammatory drugs, flufenamic acid (FFA), niflumic acid (NFA), and mefenamic acid (MFA), can stimulate two-pore potassium channels. FFA has been demonstrated to increase the outward current that is inhibited by the p.G236R mutation of KCNK9, indicating that FFA may offer a potential therapeutic benefit. In two patients with p.G236R mutations, treatment with MFA was administered without adverse reactions and showed positive effects, particularly on growth (9). However, these drugs have only been shown to have effects on the p.G236R variant and there is a lack of large-scale symptomatic cases or long-term follow-up, making it difficult to evaluate their effectiveness. Cooper et al. (10) proposed another form of treatment that may improve clinical symptoms by increasing the expression of the normal paternal allele in mice models through the inhibition of histone deacetylation. In addition to pharmaceutical therapy, early rehabilitation intervention is critical to improving the prognosis of Birk-Barel syndrome. Rehabilitation training can improve the progress in milestones while significantly improving patent's motor assessment scores in this case.

However, the diagnosis of Birk-Barel syndrome is always delayed, leading to poor treatment and rehabilitation training. In prenatal diagnosis, we emphasize multi-disciplinary evaluation of genetic diagnosis while special facial features found by B-ultrasound. With special facial features, low muscle tone, or OSA related to the disease during the infancy after birth, genetic diagnosis is also necessary done for the proband as soon as possible. Early identification of specific clinical phenotypes will help us conduct adequate familial WES analysis can help to distinguish rare unexplained syndromes.

A multi-disciplinary team of specialists should be involved for treatment and follow-up after diagnosis. And in the follow-up project should including nutrition evaluation, ophthalmology assessment, Otorhinolaryngology and gastroenterology evaluation, improvement of sleep-disordered breathing, a musculoskeletal evaluation for joint problems and the assessment of the development. Except for rehabilitation training, gene therapy may be great potential. Gene therapy, especially in the fields of neuro and cardiovascular systems, holds the most promise as a treatment alternative for inherited diseases with the advancement of the CRISPR-Cas system, base editing system, and adeno-associated virus delivery system.

## 4. Conclusion

Birk-Barel syndrome is a rare genetic disorder that poses a significant challenge to timely identification, particularly during the neonatal period, due to its non-specific clinical features. This study reports the youngest case of Birk-Barel syndrome, characterized by a KCNK9 c.710C>A p.A237D variant and severe persistent neonatal OSA. This case report expands our understanding of Birk-Barel syndrome and highlights the importance of OSA as a potential onset manifestation. Rapid WES analysis facilitated the prompt identification of the KCNK9 variant within 1 month of birth, leading to improved diagnosis and treatment outcomes. The findings of this case emphasize the importance of considering genetic variants associated with severe neonatal OSA, and suggest that neurological developmental impairments should be suspected in cases of severe recurrent OSA. The use of adequate WES assessment promotes early intervention and improved prognosis for young children with neurological disorders.

## Ethics statement

The studies involving human participants were reviewed and approved by Ethics Committee of West China Second Hospital of Sichuan University. Written informed consent to participate in this study was provided by the participants' legal guardian/next of kin. Written informed consent was obtained from the individual(s), and minor(s)' legal guardian/next of kin, for the publication of any potentially identifiable images or data included in this article.

## Author contributions

QZ, FY, and JL were the patient's physicians. QZ and ZQ reviewed the literature and contributed to manuscript drafting. ZQ and YL performed the mutation analysis. JL and YL conceptualized and designed the study, coordinated and supervised data collection, and critically reviewed the manuscript for important intellectual content. YL, FY, and JL were responsible for the revision of the manuscript for important intellectual content. All authors issued final approval for the version to be submitted.
